# Gut microbiota dysbiosis drives stroke-associated pneumonia: mechanisms and targeted therapeutic strategies

**DOI:** 10.3389/fnins.2025.1677744

**Published:** 2025-11-20

**Authors:** Jun Xiao, Jing Xia, Zhiying Chen, Weiwei Zha, Tian Xu, Xulong Chen, Xiaoping Yin

**Affiliations:** 1 Department of Neurology, Jiujiang University Affiliated Hospital, Jiujiang, Jiangxi, China; 2 The First Clinical College of Gannan Medical University, Ganzhou, Jiangxi, China; 3 School of Clinical Medical, Jiujiang University, Jiujiang, Jiangxi, China

**Keywords:** stroke-associated pneumonia, gut microbiota dysbiosis, microbiota- gut lung axis, microbiota gut brain axis, microecological therapy

## Abstract

The gut microbiota has been increasingly recognized as a central regulator of immune function, with growing research highlighting its association with the development of stroke-associated pneumonia (SAP). This review provides an overview of current research on the correlation between SAP and alterations in gut microbial composition and metabolism, with a focus on microbial imbalance, changes in key metabolites, and relevant biological mechanisms. Clinical and preclinical studies consistently report a decline in short-chain fatty acids (SCFAs)-producing bacteria, an increase in potentially harmful microbial species, reduced SCFAs levels, and elevated lipopolysaccharide (LPS) concentrations. These disturbances appear to be associated with SAP progression through the microbiota–gut–brain and microbiota–gut–lung axes by affecting immune regulation and inflammatory responses. The review also examines microbiota-targeted treatment approaches, including dietary modification, antibiotic therapy, probiotics, microbiota-regulating compounds, fecal microbiota transplantation (FMT), and respiratory microbiota transfer. A deeper understanding of how microbial disturbances are correlated with SAP may help explain the increased vulnerability to pulmonary infections following stroke and support the design of more effective, microbiota-based therapeutic strategies.

## 1 Introduction

Stroke-associated pneumonia (SAP) is one of the most common infectious complications after stroke, with an incidence of about 7 to 38% (Chaves et al., 2022). SAP is associated with higher mortality and disability rates, longer hospital stays, and worse clinical outcomes (Kaur et al., 2019). According to the Pneumonia in Stroke Consensus Group, SAP refers to pneumonia that occurs within 7 days after stroke onset in patients who are not receiving mechanical ventilation (Smith et al., 2015). Unlike community-acquired pneumonia (CAP), SAP is related to impaired oral clearance in stroke patients. Its underlying causes share similarities with hospital-acquired pneumonia (HAP) and ventilator-associated pneumonia (VAP) (Ewan et al., 2017). Three primary mechanisms may contribute to SAP development: stroke-induced immunosuppression (SIIS), an increased risk of aspiration due to swallowing difficulties, and alterations in microbial colonization. These interacting elements collectively amplify the infection risk in stroke patients.

Although SAP and aspiration pneumonia are sometimes used interchangeably, they represent distinct clinical entities. Aspiration pneumonia refers to an acute chemical lung injury caused by the aspiration of gastric contents following a aspiration event. Its symptoms develop rapidly, within minutes to hours, and typically resolve within 24–48 h (Girard and Bai, 2023). In contrast, SAP includes not only infections related to aspiration but also pneumonias resulting from stroke-induced immune dysfunction and microbial dysbiosis, which usually require antibiotic intervention and do not resolve spontaneously. Therefore, SAP represents a broader, multifactorial post-stroke pulmonary complication that cannot be fully explained by aspiration alone.

According to recent research, alterations in microbial metabolites and disturbed gut microbiota are strongly associated with the development of SAP (Xia et al., 2021). After a stroke, disruptions in gut microbiota composition can result in a decline of helpful bacteria, a surge in potentially harmful microbes, and shifts in microbial byproducts. These disturbances may weaken the gut immune defenses and could trigger broader immune dysfunction and inflammation throughout the body. Consequently, gut microbiome alterations may serve as early indicators associated with SAP, facilitating early detection (Faura et al., 2021). The gut microbiome can interacts with the central nervous system (CNS) and the lungs through microbiota–organ axes, such as the gut–brain axis and the gut–lung axis, and may be linked to the onset and progression of SAP (Wypych et al., 2019; Cryan et al., 2019). In addition, the gut microbiota and their byproducts has become a target for new treatment strategies for SAP, including dietary changes, probiotics and prebiotics, fecal microbiota transplantation (FMT), and, more recently, respiratory microbiota transplantation. These strategies exhibit promising advantages in preliminary and early clinical studies. Further research into how after stroke microbial alterations are associated with SAP could enhance understanding of the underlying mechanisms and support early diagnosis, risk prediction, and the development of personalized therapeutic strategies.

Therefore, to better understand the mechanisms and therapeutic potential of the gut microbiota in SAP, this review focuses on three key aspects (Chaves et al., 2022). The potential association between post-stroke gut microbiota dysbiosis and the development of SAP is examined, based on findings from clinical studies and preclinical models, with an emphasis on characteristic changes in microbial composition and metabolites after stroke (Kaur et al., 2019). The possible mechanisms by which the gut microbiota and its metabolites may influence or be associated with SAP pathogenesis, particularly via immune modulation through the microbiota–gut–brain and microbiota–gut–lung axes (Smith et al., 2015). Microbiota-targeted interventions are evaluated as emerging strategies for SAP treatment, including the current research progress and clinical potential of approaches such as dietary intervention, antibiotics, probiotics, FMT, and respiratory microbiota transplantation.

Relevant literature was retrieved from PubMed and Web of Science using the keywords “stroke-associated pneumonia,” “gut microbiota,” “microbiome,” “metabolites,” and “dysbiosis”. Publications in English from January 2020 to September 2025 were screened, including peer-reviewed clinical and preclinical studies assessing associations between SAP and microbiota-related features. Conference abstracts, non–peer-reviewed items, and duplicates were excluded. To preserve context, seminal pre-2020 studies were selectively cited when directly relevant, while the synthesis primarily reflects the recent 5-year literature.

## 2 Microbial changes in stroke-associated pneumonia

### 2.1 The correlation between gut dysbiosis and stroke-associated pneumonia

Following a stroke, significant changes occur in the body’s immune function. The gut microbiota has garnered increased attention as a crucial modulator of the immune response. Recent clinical studies have reported associations between the pathogenic bacterial spectrum in patients with SAP is closely related to their own gut microbiota, indicating that gut microbiota dysbiosis and bacterial translocation may be a significant factors in the development of SAP (Shim and Wong, 2018). In patients with acute post-stroke infection, more than 70% of cultivable bacteria identified in blood, sputum, and urine samples were common intestinal microorganisms, such as *Bacteroides*, *Prevotella*, and *Faecalibacterium* (Wen and Wong, 2017; Stanley et al., 2016). In cases of stroke-associated pneumonia, bacteria commonly found in positive sputum cultures include gut-resident organisms such as *Escherichia coli*, *Klebsiella pneumoniae*, *Enterobacter cloacae*, and *Enterococcus* (Kishore et al., 2018). These findings support the “gut-derived infection” hypothesis in SAP.

Evidence from preclinical model studies further support this view. In a mouse model of intracerebral hemorrhage (ICH), the lung microbiota gradually shifted toward the composition of the gut microbiota by day 7 post-stroke, indicating potential gut-to-lung bacterial transfer (Zhang et al., 2021). Experimental studies also demonstrated increased in bacterial counts in the lungs and a decrease in bacterial counts in the ileum and colon following a stroke. These localized changes in microbial distribution provide further evidence that gut microbiota dysbiosis may contribute to pulmonary infection (Zhang J. et al., 2024). Clinical studies have shown that this process allows bacteria or their components, such as lipopolysaccharide (LPS), to reach the lungs and modulate local immune responses (Błaż et al., 2024). Recent cross-sectional study reported that *Enterobacteriaceae*, including *Klebsiella pneumoniae* and *Escherichia coli*, are among the most common pathogens identified in patients with SAP (Mohapatra et al., 2024).

Collectively, current clinical and experimental findings suggest that post-stroke disruption of intestinal microbial homeostasis is associated with the translocation of gut-derived bacteria or their components, which may enhance systemic inflammation and increase susceptibility to SAP. Nevertheless, it is important to recognize that, while aspiration and SAP are sometimes discussed together, they represent overlapping but not identical processes. Aspiration resulting from post-stroke dysphagia is an important factor contributing to the development of SAP (Chang et al., 2022; Lidetu et al., 2023). Early identification of high-risk patients, combined with respiratory rehabilitation and nutritional management, can further reduce the risk of aspiration pneumonia. Studies have shown that implementing evidence-based nursing (EBP) interventions to prevent aspiration in stroke patients can significantly lower the incidence of SAP and improve clinical outcomes (Liu Z. Y. et al., 2022).

In contrast, gut–lung bacterial translocation represents an indirect pathway that may aggravate pulmonary inflammation through hematogenous or lymphatic dissemination when intestinal barrier integrity is compromised. Animal studies have demonstrated that following stroke, disruption of the intestinal barrier allows gut-derived bacteria and their products, such as LPS, to translocate to the lungs through the bloodstream or lymphatic system, where they activate pulmonary immune responses and promote inflammation (Díaz-Marugan et al., 2023; Wang et al., 2024). In contrast, clinical studies in stroke patients have reported elevated circulating LPS levels and reduced concentrations of short-chain fatty acids (SCFAs), reflecting increased gut permeability and microbial dysbiosis (Xia et al., 2021; Błaż et al., 2024). Together, these alterations disrupt gut–lung immune homeostasis and exacerbate pulmonary inflammation, thereby contributing to the development of SAP.

In summary, the aspiration pathway represents a direct route of injury, while gut–lung translocation exacerbates immune dysregulation and inflammatory responses, together constituting key mechanisms underlying the pathogenesis of SAP.

### 2.2 The composition and characteristics of gut microbiota in patients with stroke-associated pneumonia

Following a stroke, the body often experiences gut microbiota dysbiosis, characterized by a reduction in beneficial microbes and an increase in opportunistic or pathogenic bacteria. Clinical studies have shown that, at the phylum level, healthy individuals typically have gut microbiota dominated by *Bacteroidetes* and *Firmicutes* (Human Microbiome Project Consortium, 2012a; Human Microbiome Project Consortium, 2012b; Lloyd-Price et al., 2016). In contrast, stroke patients frequently exhibit higher levels of *Proteobacteria* and lower levels of *Bacteroidetes* (Benakis et al., 2016). At the genus level, SAP patients have significantly lower levels of SCFAs-producing probiotics in the gut, such as *Roseburia*, *Prevotella*, *Bacteroides*, and *Butyricicoccus*. Meanwhile, opportunistic or conditionally pathogenic bacteria, including *Enterococcus*, *Parabacteroides*, and *Corynebacterium*, tend to overgrow (Xia et al., 2021; Luo et al., 2022; Haak et al., 2021; Li Z. et al., 2023). Several studies have reported that reduced levels of SCFAs-producing bacteria are an independent predictor associated with SAP (Haak et al., 2021; Li Z. et al., 2023). A prospective cohort study in China found that SAP patients had significantly lower levels of *Roseburia* in their feces, accompanied by reduced fecal SCFAs and increased serum D-lactic acid, indicating intestinal barrier disruption (Xia et al., 2021). Another study reported that *Prevotella* abundance was negatively correlated with SAP severity and poor outcomes. The same study also showed that *Enterococcus* species promote systemic inflammation and SIIS by increasing interleukin-1 receptor antagonist (IL-1Ra) levels and reducing interferon gamma-inducible protein 10 (IP-10), thereby contributing to SAP risk (Luo et al., 2022). Notably, treatment with IL-1Ra during the acute phase of stroke can reverse peripheral innate immune suppression. In the SCIL-STROKE trial, administration of IL-1Ra reduced inflammatory markers and was associated with improved clinical outcomes in patients with ischemic stroke (Smith et al., 2018; Smith et al., 2012). Although IL-1Ra is generally regarded as an anti-inflammatory cytokine, its elevation in this context may reflect a compensatory feedback response to excessive IL-1 signaling triggered by *Enterococcus* invasion, rather than effective suppression of inflammation (Luo et al., 2022). Clinical research observations indicate that IL-1β and IL-1Ra levels rise together in the early subacute phase of stroke, implying that IL-1Ra upregulation serves as a compensatory response to IL-1–mediated inflammation rather than reflecting its complete suppression (Kotlega et al., 2025). Interestingly, some studies have noted an overall increase in *Lactobacillus* species in the gut of post-stroke patients. Among these, *Lactobacillus ruminis* was significantly enriched and positively associated with systemic inflammatory markers, suggesting a possible role in SAP-related inflammation. A similar trend was observed in patients with severe pneumonia, where the abundance of *Lactobacillales* was higher in severe cases than in milder ones, indicating that specific microbial enrichment may be linked to disease severity (Xia et al., 2021; Yamashiro et al., 2017).

Preclinical animal studies have also reported similar patterns of gut microbiota dysbiosis after stroke. These include a rise in opportunistic or conditionally pathogenic bacteria and a decline in probiotic bacteria that produce SCFAs. For example, in a *Klebsiella pneumoniae* (KP)-induced SAP mouse model, analysis of fecal samples showed a reduction in *Firmicutes* and an increase in *Actinobacteria* at the phylum level. At the genus level, the abundance of beneficial bacteria, such as *Allobaculum* and *Faecalitalea*, was decreased, while opportunistic pathogens, including *Turicibacter*, *Dietzia*, *Corynebacterium*, and *Clostridium sensu stricto* 1,were abnormally increased (Wang et al., 2024). Similarly, studies using middle cerebral artery occlusion (MCAO) mouse models have reported related changes in gut microbiota. In these models, fecal analysis revealed increased abundance of *Enterobacter* spp., *Escherichia coli*, *Shigella flexneri*, *Enterococcus faecalis*, *Staphylococcus aureus*, and *Staphylococcus sciuri* (Díaz-Marugan et al., 2023). However, inhibition of Enterobacteriaceae overgrowth in the gut did not prevent their colonization in the lungs after stroke, suggesting that other mechanisms, such as aspiration, may also contribute to the development of SAP. In another study using an ICH model, the lung microbiota on day 7 after stroke resembled the gut microbiota. The relative abundance of bacteria, including *Lactobacillus*, *Candidatus Arthromitus*, and *Escherichia coli*, was increased in both lung and intestinal tissues. These findings indicate that post-stroke pulmonary infections are associated with gut microbiota dysbiosis, which aligns with clinical observations (Zhang et al., 2021).

Collectively, both clinical and preclinical evidence indicate that stroke-induced gut dysbiosis, depletion of SCFA-producing bacteria, and compensatory immune changes are correlated with impaired intestinal immune homeostasis and heightened systemic inflammation, thereby creating conditions that favor SAP development.

Table 1 summarizes recent studies on alterations in gut microbiota and metabolites in SAP.

**Table 1:** Comparison of gut microbiota and metabolite alterations in SAP-related studies.

Studies	Subjects/Experimental models	Types of stroke	Microbiome methods	Microbiome methods	Specific microbiota	Metabolite changes
Haak et al. (2021)	Treatment group (*n* = 186); Control group (*n* = 51)	AIS or ICH	16S rRNA	↑	*Enterococcus species, Escherichia*/*Shigella species;*	Fecal butyrate ↓; trimethylamine N-oxide (TMAO) ↑
				↓	*Anaerostipe*, *Ruminococcus*, *Subdoligranulum*	
Xia et al. (2021)	SAP (*n* = 52); Controls (*n* = 136)	AIS	16S rRNA	↑	*Enterobacteriaceae*, *Erwinia*, *Enterococcaceae*, *Enterococcus*;	Fecal SCFAs ↓; serum D-lactate ↑
				↓	*Roseburia*	
Luo et al. (2022)	ICH (*n* = 64); CHD controls (*n* = 46); Healthy controls (*n* = 23)	ICH	16S rRNA	↑	*Enterococcus*, *Parabacteroides*, *Blautia*, *Lachnoclostridium*, *Acidaminococcus*;	/
				↓	*Prevotella*	
Li Z. et al. (2023)	SAP patients (*n* = 43); Controls (*n* = 92)	AIS	16S rRNA	↑	*Corynebacteriaceae*, *Corynebacterium*, *Clostridium innocuum*;	/
				↓	*Bacteroides, Coprococcus, Fusicatenibacter, Butyricicoccus, Butyricimonas, Clostridium-IVb*	
Wang et al. (2024)	SAP (*n* = 14); Sham (*n* = 10); ICH (*n* = 9)	ICH	16S rRNA	↑	*Actinobacteria*, *Turicibacter*, *Dietzia*, *Corynebacterium*, *Clostridium_sensu_stricto_1*;	Ceramides, neurotoxic quinolinic acid (QA), and Trp–serotonin–melatonin pathway ↑
				↓	*Firmicutes*, *Allobaculum*, *Faecalitalea*	
Díaz-Marugan et al. (2023)	MCAO mouse model (*n* = 249)	MACO	16S rRNA	↑	*Enterobacter* spp., *E. coli*, *Shigella flexneri*, *Enterococcus faecalis*, *Staphylococcus aureus*, *Staphylococcus sciuri*;	Hepatic bile acids (BAs) ↑; intestinal BAs and SCFAs ↓
Zhang et al. (2021)	ICH mouse model	ICH	16S rRNA	↑	*Romboutsia*, *Escherichia coli*, *Peptostreptococcaceae*	/

## 3 Potential mechanisms of gut microbiota dysbiosis in the pathogenesis of SAP

### 3.1 The microbiota–gut–brain Axis in SAP pathogenesis

Although SAP is associated with notable changes in gut microbiota and their metabolites, the specific biological pathways through which these changes influence brain function and systemic outcomes remain incompletely understood. The microbiota-gut-brain axis (MGBA) refers to the two-way communication between the CNS and the enteric nervous system (ENS) (Cryan et al., 2019). The gut microbiota, along with their SCFAs byproducts—most notably butyrate—play an essential role in facilitating gut-brain communication by acting as critical molecular messengers (Stanley et al., 2018). Under normal conditions, these molecules help maintain gut-brain communication and contribute to overall homeostasis. After stroke, three major pathways within the MGBA are disrupted (Granados-Martinez et al., 2024; Chaves et al., 2022). Autonomic nervous system (ANS) dysregulation, which affects ENS-CNS communication and influences both gut function and brain activity (Faura et al., 2021; Kaur et al., 2019). Hypothalamic–pituitary–adrenal (HPA) axis disturbed, which modifies gut barrier function, movement, and mucus production, reshaping the microbial ecosystem (Wang et al., 2022; Smith et al., 2015). Immune system dysregulation, characterized by SIIS, excessive inflammatory cytokine release, and immune cell migration. Together, these changes disrupt gut-brain communication, damage the intestinal mucosal barrier, disturb microbial balance, promote bacterial translocation, and allow microbial metabolites to enter the bloodstream—factors that may contribute to the development of SAP. Post-stroke disruption of the MGBA is reflected by reduced microbial diversity, increased abundance of opportunistic pathogens such as *Escherichia coli*, *Enterococcus*, and *Proteobacteria*, and decreased levels of beneficial bacteria like *Bacteroides*, *Prevotella*, and *Faecalibacterium* (Yin et al., 2015). In addition, the weakened intestinal epithelial barrier and lymphocyte depletion associated with SIIS allow opportunistic pathogens in the gut to colonize the lungs and other organs. Notably, suppressing the overgrowth of *Enterobacteriaceae* in the gut does not prevent their colonization in the lungs after stroke, suggesting that aspiration may contribute to this process (Díaz-Marugan et al., 2023).

However, direct evidence of gut-to-lung bacterial translocation in humans remains limited and warrants further investigation.

### 3.2 The microbiota–gut–lung axis in SAP pathogenesis

Recent studies have shown that the lung microbiota shifts to resemble the gut microbiota following stroke, indicating potential microbial exchange via the gut-lung axis (Dang and Marsland, 2019). Although research on the gut-lung axis in SAP is still limited, current evidence indicates that gut microbes may translocate to the lungs in patients with SAP. This observation suggests that the gut microbiome may be associated with the onset and progression of SAP through multiple pathways. Gut microbiota metabolites shape the lung microbial ecosystem and modulate pulmonary immunity by altering the local immune environment. Similar gut–lung interactions have been described in other respiratory diseases, including chronic obstructive pulmonary disease, bronchial asthma, and other chronic inflammatory conditions, as well as in viral pneumonia and bronchiectasis (Narayana et al., 2023; Perdijk et al., 2024; Wang et al., 2023).

Post-stroke stress responses triggered by the HPA axis and the autonomic nervous system, together with microbiota dysbiosis, lead to increased intestinal epithelial cell death and disruption of the mucus layer and tight junctions. These changes result in increased intestinal permeability (Prame Kumar et al., 2025). Once the intestinal barrier is compromised, gut microbiota and their byproducts can migrate to the lungs and additional organs via the circulatory or lymphatic pathways (Granados-Martinez et al., 2024). In addition, stroke induces systemic immunosuppression (Faura et al., 2021), which allows opportunistic pathogens, such as *Enterobacteriaceae*, to expand in the absence of effective local immune defense. These pathogens can activate inflammatory pathways in alveolar macrophages and epithelial cells, leading to innate immune responses and activating the nuclear factor kappa B (NF-κB) signaling pathway. As a result, pro-inflammatory cytokines such as tumor necrosis factor-alpha (TNF-*α*) and interleukin-1β (IL-1β) are released, which ultimately lead to lung inflammation (Díaz-Marugan et al., 2023).

By secreting soluble substances and metabolites such as SCFAs, peptidoglycans, and LPS, the gut microbiota can modulate lung immune response. These microbial products are collectively known as microbe-associated molecular patterns (MAMPs), have important immunoregulatory properties. Pattern recognition receptors (PRRs), such as Toll-like receptors (TLRs) and Nod-like receptors (NLRs), allow host innate immune cells to identify MAMPs (Wypych et al., 2019). Furthermore, one study reported that modifying the gut microbiota to increase SCFAs levels had a protective effect on lung inflammation (Mao et al., 2020). These findings highlight the gut-lung axis’s crucial role in maintaining lung immune balance.

Beyond molecular signaling, the gut-lung axis may facilitate immune crosstalk through the migration of circulatory immune cells. In one animal study, the circulatory systems of two mice were connected. One mouse received an intraperitoneal injection of interleukin-25 (IL-25), resulting in the activation of intestinal group 2 innate lymphoid cells (ILC2s). This treatment activated ILC2s, which were later detected not only in the lungs of the treated mouse but also in the lungs of the connected mouse (Huang et al., 2018). This finding suggests that immune cells activated in the gut can travel through the circulation and influence immune function in distant organs such as the lungs. SIIS further impairs local lung immunity by reducing the phagocytic capacity of alveolar macrophages, increasing the risk of SAP (Samary et al., 2018). Exogenous immune cells play a protective role in preventing lung inflammation. However, some gut-derived immune cells can have harmful effects. For example, *γ*δ T cells originating in the intestine can migrate to the lungs via the bloodstream and produce cytokines such as interleukin-6 (IL-6), IL-22, TNF-*α*, and IFN-γ, which exacerbate pulmonary inflammation (Shichita et al., 2009). Inhibiting the migration of intestinal γδ T cells to the brain and lungs has been associated with reduced ischemic brain injury and decreased severity of SAP (Xie B. et al., 2023).

These findings suggest the gut-lung axis may play a role in SAP pathophysiology, though further studies are required to clarify the exact connection.

### 3.3 Impact of microbial metabolites on the pathogenic mechanisms of SAP

Post-stroke microbiome shifts alter metabolite levels, affecting SAP development via immune modulation and intestinal barrier integrity. Recent studies have shown that gut dysbiosis not only increases intestinal permeability and chronic inflammation but also contributes to inflammatory responses in the lungs.

LPS, a Gram-negative bacterial outer membrane element, strongly triggers immune reactions. Evidence suggests that stroke can disrupt the integrity of the intestinal barrier, allowing bacterial components such as LPS to enter the bloodstream. This increase in circulating LPS is associated with stroke severity (Azzoni and Marsland, 2022; Swidsinski et al., 2012; Hakoupian et al., 2021). Once in the bloodstream, LPS can impair the pulmonary immune barrier through the gut-lung axis and triggering pulmonary inflammation (Xie X. et al., 2023). For example, LPS promotes the recruitment of neutrophils into the lungs, stimulates macrophages to release inflammatory cytokines, and causes acute lung injury (Grommes and Soehnlein, 2011). Pathogen-associated molecular patterns (PAMPs), including LPS and flagellin, interact with PRRs such as TLR4, TLR5, and NLRP3. This activates the NF-κB and inflammasome pathways, resulting in the production of pro-inflammatory cytokines such as IL-1β, interleukin-6 (IL-6), and TNF-α, and contributes to peripheral immune dysregulation and lung tissue damage (Kawai et al., 2024; Özçam and Lynch, 2024; Le et al., 2023). In animal models of stroke, intratracheal administration of LPS has been shown to successfully induce SAP-like inflammatory responses (Wang et al., 2024), demonstrating the pathogenic potential of LPS in post-stroke pneumonia. Although this study did not directly examine gut-to-lung exposure to LPS may contribute to SAP pathogenesis.

SCFAs, including acetate, propionate, and butyrate, are major metabolites formed through gut microbial fermentation of dietary fiber. These metabolites possess anti-inflammatory and immunomodulatory properties (Tedelind et al., 2007). SCFAs play a crucial role in enhancing host immune function, inhibiting the growth of harmful bacteria, and maintaining the integrity of gut and lung epithelial barriers (Fukuda et al., 2011). For example, SCFAs can promote the recruitment of immune cells to the airways and lungs (Dang and Marsland, 2019). They also influence T-cell differentiation into Th1 and Th17 subsets while inducing IL-10 production in regulatory T cells (Treg). These effects are associated with reduced concentrations of pro-inflammatory cytokines IL-6, increases IL-10 levels, and helps suppress intestinal and systemic inflammation (Tedelind et al., 2007; Park et al., 2015). Following a stroke, SCFAs levels are reduced due to a decline in anaerobic fiber-fermenting bacteria in the gut microbiota (Díaz-Marugan et al., 2023; Tan et al., 2021). Among SCFAs, butyrate appears to have the most significant effect and is positively associated with reduced risk of lower respiratory tract infections (Haak et al., 2018; Vinolo et al., 2011; Chen et al., 2019). The anti-inflammatory action of butyrate is linked to the inhibition of the NF-κB signaling pathway, increased IL-10 production, and reduced production of pro-inflammatory cytokines, such as IL-12 and IFN-*γ* (Kotlyarov, 2022). SCFAs also support pulmonary immune defense by promoting the expansion of myeloid progenitor cells in the in bone marrow, enhancing the phagocytic activity of alveolar macrophages, and reducing pro-inflammatory activity in macrophages (Scott et al., 2018; Wu et al., 2020; Li T. et al., 2023; Engel et al., 2015). Butyrate supplementation has been shown to selectively activate oxidative phosphorylation (OXPHOS) and lipid metabolism in macrophages, enhancing their anti-inflammatory functions (Scott et al., 2018). Furthermore, a SAP model study show that bacterial clearance in the lungs can be enhanced through phagocytosis by alveolar macrophages, which is promoted by extracellular vesicles released from bone marrow mesenchymal stem cells (BM-MSCs), potentially helping to prevent the onset of SAP (Li T. et al., 2023; Engel et al., 2015). Therefore, reduced SCFAs levels after stroke may disrupt the intestinal-pulmonary mucosal immune balance and contribute to the development or worsening of SAP.

In summary, stroke alters the metabolic profile of the gut microbiota by disrupting the intestinal barrier and activating systemic stress responses. These metabolites, particularly LPS and SCFAs, can promote or worsen pulmonary infection by influencing inflammatory cytokine production, immune cell function, and the integrity of epithelial barriers.

## 4 Gut microbiota–targeted therapies in SAP

### 4.1 Dietary interventions

Approximately 75% of stroke patients experience dysphagia (Labeit et al., 2023). Early dietary adjustments, such as the use of a nasogastric tube (NGT), fasting, or fluid restriction (nil per os, NPO), are commonly used to reduce the incidence of chest infections in stroke patients with dysphagia (Sørensen et al., 2013; Carnaby et al., 2006). Restoring oral feeding is crucial even if research indicates that early NGT usage may reduce the risk of SAP, mortality, or poor functional outcomes. Prolonged NGT use may impair swallowing function in older patients and increase the likelihood of aspiration (Kalra et al., 2016). A study on dietary management in stroke patients found that even with NPO measures, 60% of patients with severe dysphagia developed SAP (Teuschl et al., 2018). A retrospective study in Australia reported that 37% of patients with NGTs developed respiratory infections, compared to only 5% of those without NGT (Brogan et al., 2014). Clinical data also suggest that in patients with swallowing difficulties, manual oral feeding reduces the risk of aspiration pneumonia by about 40% compared to NGT feeding (Yuen et al., 2022). Patients who were denied oral eating throughout their hospital stay experienced longer treatment durations, worse swallowing results, and a greater risk of aspiration pneumonia than those who were allowed to eat earlier (Maeda et al., 2016). Additionally, studies have demonstrated that reintroducing oral feeding after enteral nutrition can impact both the oral and gut microbiota. Oral feeding increases the abundance of *Actinobacteria* and decreases the abundance of *Proteobacteria* in the oral cavity at the phylum level. Such as, *Verrucomicrobia* levels in the gut decline following the restart of oral feeding. At the species level, the abundance of *Lactobacillales*, *Streptococcaceae*, *Streptococcus*, *Granulicatella*, and *Streptococcus* sp. increases. These bacteria help restore the intestinal mucosal barrier and produce short-chain fatty acids (SCFAs), which may lower the risk of SAP (Katagiri et al., 2019). In summary, early resumption of oral feeding may be a promising strategy to reduce the incidence of SAP.

Furthermore, modifications to the structure of the food can have a significant impact on the gut microbiota composition and the synthesis of fermentation products, such as SCFAs and phytochemicals (Wilson et al., 2020). Consuming a high-calorie, low-fiber diet can temporarily weaken gut and immune defenses, increasing the risk of infection (Siracusa et al., 2023). A high-calorie diet may disrupt the Th17/Treg balance, worsen LPS-induced pneumonia, and decrease gut microbiota that generate SCFAs, such as those from *Lactobacillaceae*, *Muribaculaceae*, and *Lactobacillus* (Liu H. et al., 2022). In a study using MACO mice, a ketogenic diet (KD) was found to worsen neurological deficits after stroke. Microbiota changes were also observed: at the phylum level, *Firmicutes* rose in relative abundance, while *Bacteroidetes* and *Proteobacteria* declined. At the family level, the proportion of *Prevotellaceae*, a key SCFA-producing group, decreased by more than fivefold in mice receiving KD, despite an increase in *Firmicutes* (Zharikova et al., 2025). This reduction in *Prevotellaceae* may contribute to a higher risk of SAP. A high-salt diet over an extended period has also been shown to delay brain healing following ICH, impair macrophage function, and increase the relative abundance of harmful gut bacteria (Lin et al., 2023). In addition, a high-fat diet promotes the production of interleukin-17A (IL-17A) by γδ T cells and raises systemic IL-17A levels by altering the microbiota, potentially intensifying the inflammatory response (Sonomoto et al., 2023). Supplementation with micronutrients, such as vitamin E and vitamin D, may provide additional therapeutic benefits in patients with SAP (Shen and Zhan, 2020; Sheerah et al., 2018). Therefore, a diet rich in fiber, low in salt and fat, sufficient in calories, and supplemented with essential micronutrients—while avoiding high-calorie and ketogenic diets—may help restore gut microbiota balance, reduce post-stroke inflammation, and improve patient outcomes.

### 4.2 Antibiotic

The use of antibiotics following stroke to reduce the incidence of SAP represents a promising preventive strategy, although large-scale clinical trials have yet to confirm its efficacy (Westendorp et al., 2015; Kalra et al., 2015; De Jonge et al., 2020; De Jonge et al., 2022). It is important to note, however, that antibiotics can disrupt the beneficial gut microbiota, potentially exacerbating post-stroke dysbiosis and inflammatory responses (Ghelani et al., 2021). Selective digestive decontamination (SDD) is a commonly employed prophylactic antibiotic method in the care of severely ill patients, has been shown to effectively reduce the incidence of VAP and postoperative gastrointestinal pneumonia (Hammond et al., 2022; Janssen et al., 2021). Furthermore, some studies have demonstrated that SDD can decrease the colonization of aerobic gram-negative bacteria (AGNB) and lower the incidence of pneumonia following acute stroke (Gosney et al., 2006). These findings suggest that SDD may help reduce the risk of SAP, although its long-term effects on gut microbial ecology require further evaluation.

The use of broad-spectrum antibiotics, such as meropenem, gentamicin, and vancomycin, leads to significant changes in gut microbiota composition, including an increase in *Enterobacteriaceae* and other potential pathogens, and a decrease and a decline in species that produce SCFAs, including butyrate, and beneficial bacteria like *Bifidobacteria* (Palleja et al., 2018). The loss of SCFAs-producing bacteria reduces the availability of SCFAs, which may impair the bactericidal function of inflammatory macrophages and weaken pulmonary immune defenses, thereby increasing the risk of pneumonia (Dörner et al., 2024).

New-generation precision antibiotics, such as lolamicin, or targeted antimicrobial approaches may control pulmonary infections more effectively while minimizing disruption to the overall gut microbiota (Muñoz et al., 2024). Additionally, some newer broad-spectrum antibiotics have exhibited favorable properties. Preclinical evidence from animal models indicates that omadacycline, a novel aminomethylcycline-class tetracycline, causes less disturbance to gut microbial composition than vancomycin (Leahy et al., 2022). In contrast, clinical and *in vitro* colon model data suggest that omadacycline alters the gut microbiota more extensively than moxifloxacin and vancomycin, although it does not promote *Clostridioides difficile* infection (Jo et al., 2023; Moura et al., 2019). Beyond its antimicrobial activity, experimental studies have shown that omadacycline also exhibits anti-inflammatory effects. It decreases macrophage-driven pro-inflammatory cytokine and chemokine release—such as TNF-*α*, IL-1β, IL-6, CXCL-1, CXCL-2, and matrix metalloproteinase-9 (MMP-9)—and attenuates neutrophil infiltration into the lungs, thereby mitigating lung injury and infection (Sanders and Beringer, 2024). Current clinical evidence and treatment guidelines do not recommend tetracyclines, including omadacycline, as first-line agents for the management of SAP. They are used only as an alternative or add-on in selected situations. Given their potential anti-inflammatory effects and limited impact on the gut microbiota, cautious exploratory use in SAP may be considered after careful assessment of indications and risks.

In conclusion, optimizing antibiotic strategies to maintain gut microbiota balance may provide new approaches for preventing and managing SAP. Future studies should aim to elucidate the specific effects of various antibiotics on the gut microbiome and explore microbiota-targeted, personalized antimicrobial approaches to achieve more precise and safer strategies for SAP treatment.

### 4.3 Probiotics and microbial-based therapeutics

The gut microbiome plays a crucial role in preventing post-stroke complications and pulmonary diseases through its association with the regulation of host immune responses, hormone secretion, and metabolic processes (Zhang J. et al., 2024; Ma et al., 2022). Probiotics, live microbes that provide health advantages when consumed in sufficient quantities, represent a promising microbiota-based intervention strategy for SAP. According to recent research, the use of probiotics in conjunction with antibiotics has been associated with a lower risk of respiratory tract infections (Merenstein et al., 2024). Although direct evidence supporting the function of probiotics in preventing SAP is currently lacking, existing research has demonstrated that probiotics can decrease the incidence of VAP in critically ill patients in intensive care units (Li C. et al., 2022). In addition, certain microbiota-derived products, such as postbiotics, may serve as promising alternatives to probiotics. Studies have reported that postbiotic supplementation can improve inflammatory cytokine profiles and oxidative stress markers in patients with cerebrovascular events and may potentially reduce the risk of pneumonia (Rahimi et al., 2024). These findings suggest that probiotics and postbiotics hold therapeutic potential in the prevention and management of SAP, warranting further investigation.

### 4.4 FMT

FMT entails the introduction of minimally treated fecal matter from a healthy donor into the digestive system of a recipient, and is used to treat various diseases associated with gut microbiota dysbiosis, such as inflammatory bowel disease, Parkinson disease, autism, and stroke (Porcari et al., 2023; Hou et al., 2025; Hediyal et al., 2024). Recent studies have suggested that FMT may represent a potential therapeutic strategy for stroke and related complications (Hediyal et al., 2024). Although direct evidence supporting the efficacy of FMT in treating SAP is currently lacking, animal studies have shown that FMT can restore pulmonary cellular responses and improve outcomes in acute lung injury and *Pseudomonas aeruginosa*-induced lung infections (Dessein et al., 2020). Moreover, FMT has been demonstrated as a potential approach to modulate the gut microbiota in animal models. Studies have shown that FMT increases the abundance of butyrate-producing bacteria, such as *Lachnospiraceae*, *Ruminococcaceae*, *Ruminococcus*, and *Oscillospiraceae*, while lowering LPS and pro-inflammatory cytokine levels in ischemic mice, including IL-6, TNF-*α*, and IL-1β (Wang et al., 2021). Transplantation with gut microbiota enriched in SCFAs, particularly butyrate, may help alleviate the SCFAs deficiency observed after stroke (Chen et al., 2019). Specific bacterial strains such as *Faecalibacterium prausnitzii* and *Roseburia intestinalis* have been shown to correlate with enhance Treg populations and promote the expression of anti-inflammatory cytokines, thereby improving intestinal epithelial integrity and reducing mucosal lymphocyte infiltration. These gut bacteria exhibit anti-inflammatory properties and are seen as viable candidates for FMT-based treatments (Shen et al., 2022; Touch et al., 2022). While these findings offer valuable insights into the potential application of FMT in SAP, they are primarily based on animal models. Further investigations, including randomized controlled trials, are warranted to validate the therapeutic efficacy and safety of FMT in SAP, and to elucidate its mechanisms through the gut–lung and gut–brain axes.

### 4.5 Respiratory microbiota transplantation

Restoring normal upper respiratory tract microbiota has been associated with reduced incidence of CAP, HAP, and VAP by improving resistance to pathogen colonization and enhancing immune defense (Thibeault et al., 2021). Evidence from animal studies suggests that restoration of the upper respiratory microbiota may enhance immune responses in the lungs. Specifically, certain commensal strains such as *Staphylococcus epidermidis*, and some *S. aureus isolates*, have been shown to increase the phagocytic capacity of lung macrophages through the nucleotide-binding oligomerization domain-containing protein 2 (Nod2) and granulocyte–macrophage colony-stimulating factor (GM-CSF) signaling pathways. Additionally, IL-17A has been shown to promote the production of GM-CSF, which may further support this immunomodulatory mechanism (Brown et al., 2017). Nasal administration of Lactobacillus and other probiotics has been found to regulate innate immune responses in the respiratory tract. *Lactobacillus* species can boost the mucosal immune system and provide defense against *Streptococcus pneumoniae* infection (Li Z. et al., 2022). Moreover, extracellular polysaccharides from *Lactobacillus plantarum WXD301*, a commensal bacterium found in the lungs, have been shown to enhance local mucosal immunity and improve defense against *Pseudomonas aeruginosa* infection (Zhang H. et al., 2024). These findings suggest that respiratory microbiota transfer might represent a promising approach for combating pulmonary infections.

These findings indicate that transplanting respiratory microbiota could effectively combat pulmonary infections. Although no direct experimental or clinical evidence supports its application for SAP treatment or prevention, this approach may help restore microbiota imbalance after stroke. Therefore, respiratory microbiota transplantation could represent a potential therapeutic strategy for preventing or treating SAP, pending further preclinical and clinical validation.

## 5 Conclusions and future perspectives

**Figure 1:**
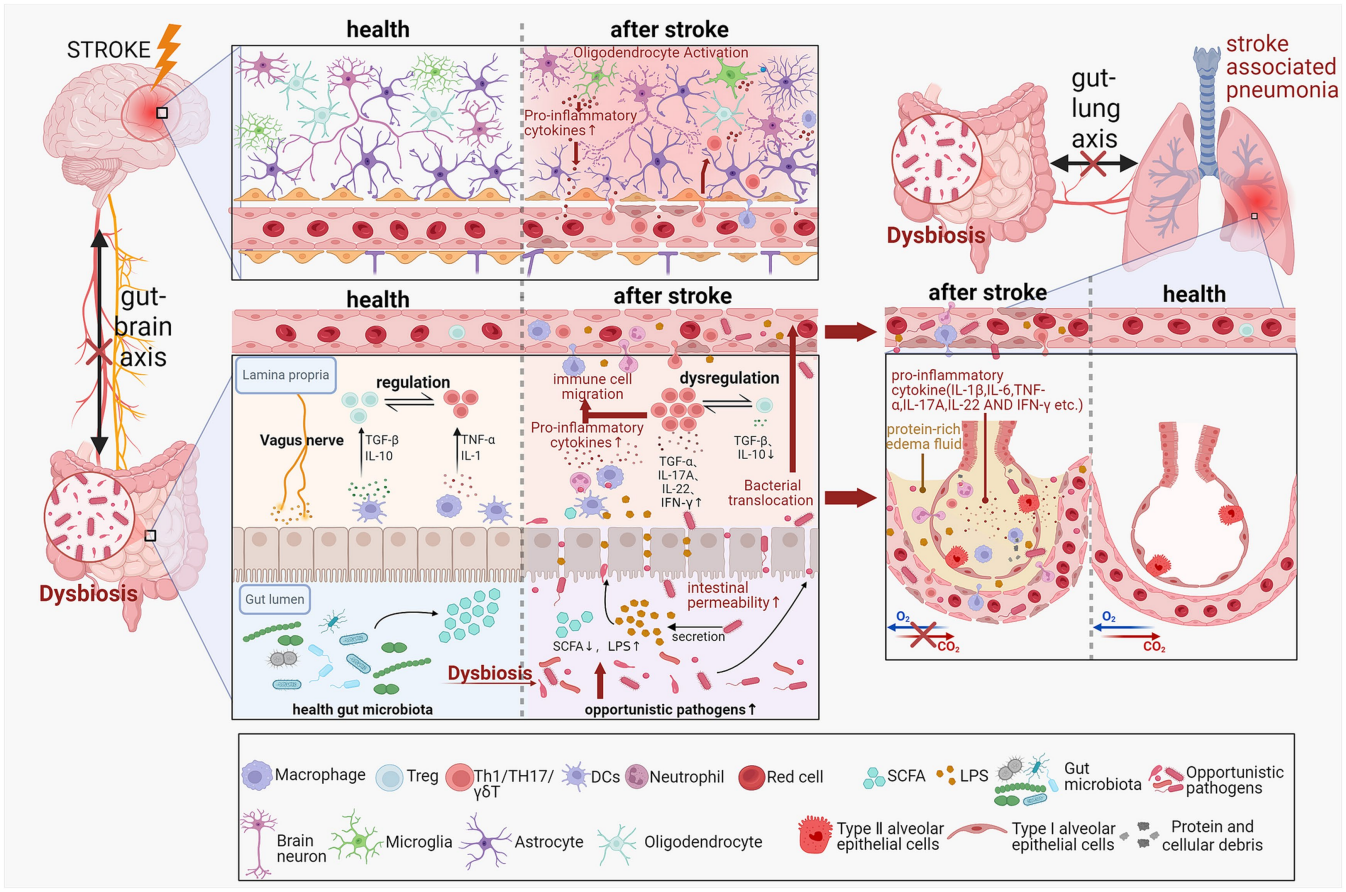
Mechanisms of gut microbiota dysbiosis in the pathogenesis of SAP. The development of SAP involves the brain, gut, and lungs. Disrupted microbiota can play a critical role in SAP pathogenesis through the microbiota–gut–brain axis and the microbiota–gut–lung axis, as well as via circulatory, immune, and neural pathways. Created with BioRender.com.

The gut microbiota and its metabolites, such as SCFAs and LPS, are crucial in modulating immune, responses, local and systemic inflammation, and maintaining epithelial barrier integrity (these mechanisms are summarized in Figure 1). Although SIIS and dysphagia are recognized as the main contributors to SAP, growing evidence suggests that opportunistic pathogens frequently detected in sputum and lower respiratory tract secretions of SAP patients mainly originate from the gut. This highlights the key role of the microbiota–gut–lung axis in the pathogenesis of SAP. Stroke-related impairment of intestinal barrier function, combined with immune imbalance in the lungs, may promote the translocation of gut bacteria and their metabolites. Gut-derived opportunistic organisms and microbial components may translocate across a weakened intestinal barrier, such as LPS; however, direct human evidence remains limited. Once in systemic circulation, these elements may migrate to pulmonary tissue, where they contribute to localized inflammatory responses. These processes can initiate or worsen the development of SAP. Further research is needed to clarify the mechanisms by which gut-derived microbes and their metabolites contribute to lung infection after stroke. A deeper insight into these pathways could aid in creating microbiota-focused approaches to prevent or mitigate SAP and enhance stroke patient recovery.

**Figure 2:**
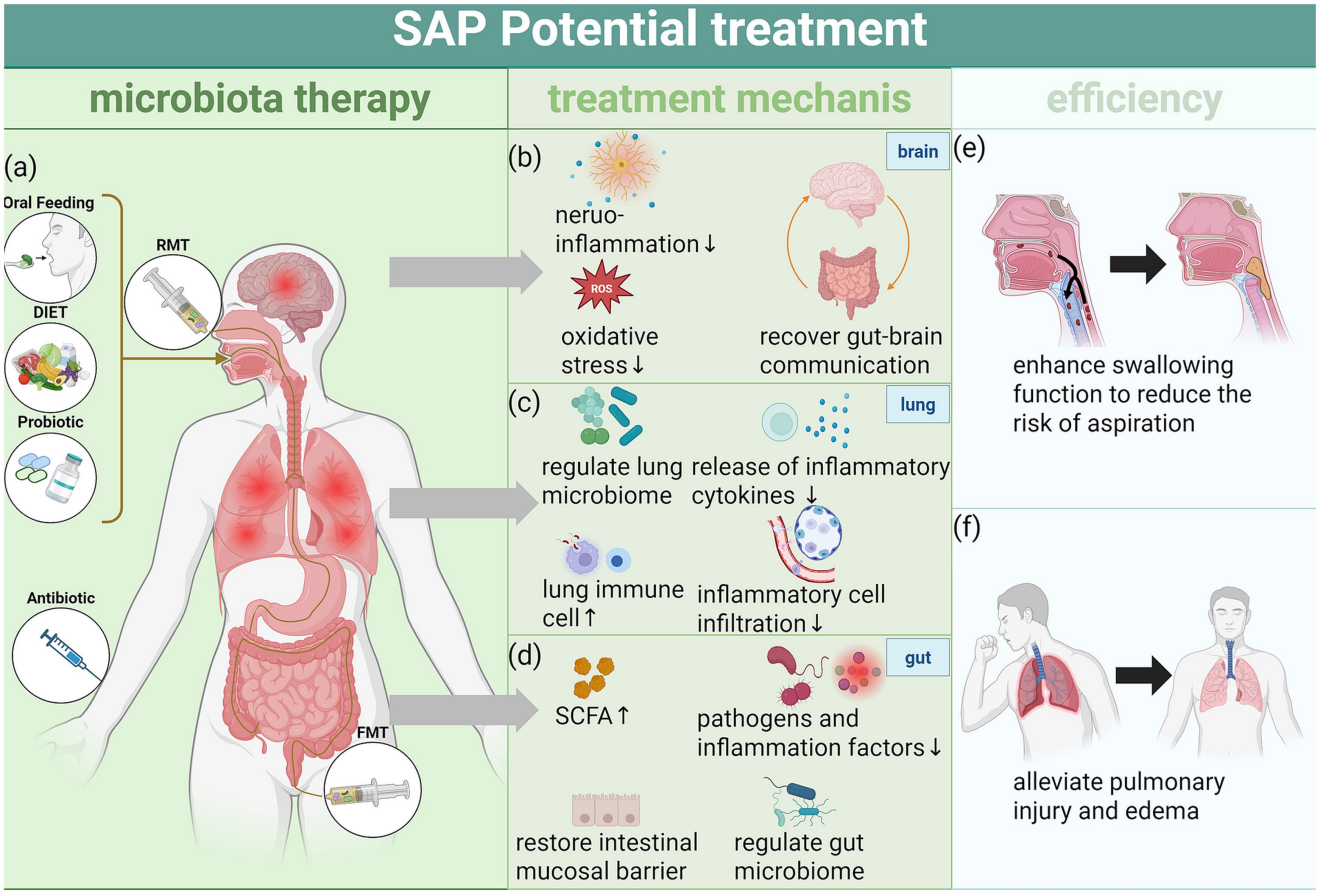
Microbiome therapy for SAP. **(a)** Current microbial therapies for SAP include dietary interventions, antibiotics, probiotics, FMT, and respiratory microbiota transplantation, administered via oral, intravenous, or endoscopic routes. **(b)** In the brain, microbial therapies can alleviate neuroinflammation and oxidative stress, thereby restoring gut–brain axis communication and improving gut microbial balance. **(c)** In the lungs, they help regulate the pulmonary microbiota, increase immune cell populations, promote the release of anti-inflammatory factors, reduce inflammatory cell infiltration, and lower pulmonary inflammation. **(d)** These therapies also promote SCFA production, repair the intestinal mucosal barrier, inhibit pathogenic bacteria, reduce pro-inflammatory cytokines, and modulate gut microbial composition, thereby suppressing intestinal inflammation, reducing the risk of intestinal leakage, and preventing bacterial translocation. **(e,f)** Overall, microbial therapies alleviate pulmonary inflammation and edema through multiple mechanisms, improve swallowing function, reduce the risk of aspiration, and effectively contribute to the treatment and prevention of SAP. Created with BioRender.com.

Although antibiotics are still the main treatment for SAP, their wide use in practice is linked to increasing antibiotic resistance, and the development of new antibiotics remains difficult. In this context, regulating the gut microbiota (treatment strategies summarized in Figure 2) has emerged as a promising therapeutic approach. Interventions such as novel antibiotics, probiotics, prebiotics, postbiotics, FMT, and respiratory microbiota transplantation may help improve gut microbiota composition and function, thereby alleviating the pathological progression of SAP. Evidence from animal studies and early clinical studies suggests potential benefit, but more high-quality trials in humans are still needed. Because many of these methods involve regulated drugs or biologic products, their safety, effectiveness, dosing, and cost should be assessed carefully before routine use. In contrast, dietary intervention offers a simple, safe, and widely accepted alternative with strong potential to improve gut microbiota dysbiosis and alleviate SAP symptoms. Restoring oral intake can improve swallowing function and lower the risk of aspiration, while optimizing dietary structure may help restore gut microbiota balance, enhance SCFAs production, and support immune barrier repair. Nevertheless, the early reintroduction of oral feeding carries a risk of aspiration, which may worsen pneumonia or lead to other serious complications. In addition, features of the gut microbiota and its metabolites show promise as early diagnostic and dynamic monitoring biomarkers for SAP, and could help stratify high-risk patients and guide individualized management. However, clinical use will require standardized testing methods, validated thresholds, and external confirmation in prospective cohorts and intervention studies.

Prevention and treatment of SAP remain key clinical priorities after stroke. This review highlights the potential roles of the microbiota and its metabolites in SAP. While SIIS and dysphagia-related aspiration are the main triggers, gut-derived microbes and metabolites (such as LPS, SCFAs) may enter the blood or lymph when the intestinal barrier is impaired and may amplify lung inflammation. Based on current evidence, we propose a stratification framework centered on “clinical risk—microbiota and metabolites—barrier and immunity.” Using graded assessment and dynamic monitoring, clinicians could rapidly identify high-risk patients and their “SAP microbiome risk phenotypes,” and then apply personalized microbiome-focused interventions—such as diet and nutrition optimization, probiotics/microecologic products, and FMT or Respiratory Microbiota Transplantation under strict indications—to ease stroke-related immunosuppression, help restore the gut barrier, and potentially reduce lung inflammatory burden and SAP risk. In parallel, antimicrobial stewardship, oral care, airway management, and swallowing management should remain the foundation and work in synergy with the above measures. It should be emphasized that direct human evidence for “gut-to-lung transfer” is still limited, key biomarkers and decision thresholds require validation, and clinical evidence for routine use of microbiome interventions is lacking. Future work should use standardized sampling and integrate multi-omics with metabolomics, strengthen safety oversight and access, and advance microbiome-guided precision prevention and treatment to improve post-stroke outcomes.

## References

[ref1] Azzoni R. Marsland B. J. (2022). The lung-brain axis: a new frontier in host-microbe interactions. Immunity 55, 589–591. doi: 10.1016/j.immuni.2022.03.015, PMID: 35417673

[ref2] Benakis C. Brea D. Caballero S. Faraco G. Moore J. Murphy M. . (2016). Commensal microbiota affects ischemic stroke outcome by regulating intestinal γδ T cells. Nat. Med. 22, 516–523. doi: 10.1038/nm.4068, PMID: 27019327 PMC4860105

[ref3] Ma P. J. Wang M. M. Wang Y. (2022). Gut microbiota: a new insight into lung diseases. Biomed. Pharmacother. 155:113810. doi: 10.1016/j.biopha.2022.113810 36271581

[ref4] Błaż M. Natorska J. Bembenek J. P. Członkowska A. Ząbczyk M. Polak M. . (2024). Elevated lipopolysaccharide level is largely driven by time since symptom onset in acute ischemic stroke: the impact on clinical outcomes. J. Thromb. Haemost. 22, 3161–3171. doi: 10.1016/j.jtha.2024.06.028, PMID: 39122194

[ref5] Brogan E. Langdon C. Brookes K. Budgeon C. Blacker D. (2014). Respiratory infections in acute stroke: nasogastric tubes and immobility are stronger predictors than dysphagia. Dysphagia 29, 340–345. doi: 10.1007/s00455-013-9514-5, PMID: 24445382

[ref6] Brown R. L. Sequeira R. P. Clarke T. B. (2017). The microbiota protects against respiratory infection via GM-CSF signaling. Nat. Commun. 8:1512. doi: 10.1038/s41467-017-01803-x, PMID: 29142211 PMC5688119

[ref7] Carnaby G. Hankey G. J. Pizzi J. (2006). Behavioural intervention for dysphagia in acute stroke: a randomised controlled trial. Lancet Neurol. 5, 31–37. doi: 10.1016/S1474-4422(05)70252-0, PMID: 16361020

[ref9] Chang M. C. Choo Y. J. Seo K. C. Yang S. (2022). The relationship between dysphagia and pneumonia in acute stroke patients: a systematic review and meta-analysis. Front. Neurol. 13:240. doi: 10.3389/fneur.2022.834240, PMID: 35370927 PMC8970315

[ref10] Chaves M. L. Gittins M. Bray B. Vail A. Smith C. J. (2022). Variation of stroke-associated pneumonia in stroke units across England and Wales: a registry-based cohort study. Int. J. Stroke 17, 155–162. doi: 10.1177/17474930211006297, PMID: 33724106 PMC8821977

[ref11] Chen R. Xu Y. Wu P. Zhou H. Lasanajak Y. Fang Y. . (2019). Transplantation of fecal microbiota rich in short chain fatty acids and butyric acid treat cerebral ischemic stroke by regulating gut microbiota. Pharmacol. Res. 148:104403. doi: 10.1016/j.phrs.2019.104403, PMID: 31425750

[ref12] Cryan J. F. O’Riordan K. J. Cowan C. S. M. Sandhu K. V. Bastiaanssen T. F. S. Boehme M. . (2019). The microbiota-gut-brain axis. Physiol. Rev. 99, 1877–2013. doi: 10.1152/physrev.00018.2018 31460832

[ref13] Dang A. T. Marsland B. J. (2019). Microbes, metabolites, and the gut–lung axis. Mucosal Immunol. 12, 843–850. doi: 10.1038/s41385-019-0160-6, PMID: 30976087

[ref14] De Jonge J. C. van de Beek D. Lyden P. Brady M. C. Bath P. M. van der Worp H. B. (2022). Temporal profile of pneumonia after stroke. Stroke 53, 53–60. doi: 10.1161/STROKEAHA.120.032787, PMID: 34517764 PMC8700305

[ref15] De Jonge J. C. Woodhouse L. J. Reinink H. van der Worp H. B. Bath P. M. PRECIOUS investigators (2020). Precious: prevention of complications to improve outcome in elderly patients with acute stroke-statistical analysis plan of a randomised, open, phase III, clinical trial with blinded outcome assessment. Trials 21:884. doi: 10.1186/s13063-020-04717-0, PMID: 33106180 PMC7586648

[ref16] Dessein R. Bauduin M. Grandjean T. Le Guern R. Figeac M. Beury D. . (2020). Antibiotic-related gut dysbiosis induces lung immunodepression and worsens lung infection in mice. Crit. Care 24:611. doi: 10.1186/s13054-020-03320-8, PMID: 33076936 PMC7574210

[ref17] Díaz-Marugan L. Gallizioli M. Márquez-Kisinousky L. Arboleya S. Mastrangelo A. Ruiz-Jaén F. . (2023). Poststroke lung infection by opportunistic commensal bacteria is not mediated by their expansion in the gut microbiota. Stroke 54, 1875–1887. doi: 10.1161/STROKEAHA.123.042755, PMID: 37226775 PMC10289215

[ref18] Dörner P. J. Anandakumar H. Röwekamp I. Fiocca Vernengo F. Millet Pascual-Leone B. Krzanowski M. . (2024). Clinically used broad-spectrum antibiotics compromise inflammatory monocyte-dependent antibacterial defense in the lung. Nat. Commun. 15:2788. doi: 10.1038/s41467-024-47149-z, PMID: 38555356 PMC10981692

[ref19] Engel O. Akyüz L. da Costa Goncalves A. C. Winek K. Dames C. Thielke M. . (2015). Cholinergic pathway suppresses pulmonary innate immunity facilitating pneumonia after stroke. Stroke 46, 3232–3240. doi: 10.1161/STROKEAHA.115.008989, PMID: 26451017

[ref20] Ewan V. Hellyer T. Newton J. Simpson J. (2017). New horizons in hospital acquired pneumonia in older people. Age Ageing 46, 352–358. doi: 10.1093/ageing/afx029, PMID: 28338911

[ref21] Faura J. Bustamante A. Miró-Mur F. Montaner J. (2021). Stroke-induced immunosuppression: implications for the prevention and prediction of post-stroke infections. J. Neuroinflammation 18:127. doi: 10.1186/s12974-021-02177-0, PMID: 34092245 PMC8183083

[ref22] Fukuda S. Toh H. Hase K. Oshima K. Nakanishi Y. Yoshimura K. . (2011). Bifidobacteria can protect from enteropathogenic infection through production of acetate. Nature 469, 543–547. doi: 10.1038/nature09646, PMID: 21270894

[ref23] Ghelani D. P. Kim H. A. Zhang S. R. Drummond G. R. Sobey C. G. De Silva T. M. (2021). Ischemic stroke and infection: A brief update on mechanisms and potential therapies. Biochem. Pharmacol. 193:114768. doi: 10.1016/j.bcp.2021.114768, PMID: 34543657

[ref24] Girard V. Bai A. D. (2023). Aspiration pneumonia. Can. Med. Assoc. J. 195:E1417. doi: 10.1503/cmaj.230628, PMID: 37871952 PMC10593196

[ref25] Gosney M. Martin M. V. Wright A. E. (2006). The role of selective decontamination of the digestive tract in acute stroke. Age Ageing 35, 42–47. doi: 10.1093/ageing/afj019, PMID: 16364933

[ref26] Granados-Martinez C. Alfageme-Lopez N. Navarro-Oviedo M. Nieto-Vaquero C. Cuartero M. I. Diaz-Benito B. . (2024). Gut microbiota, bacterial translocation, and stroke: current knowledge and future directions. Biomedicine 12:2781. doi: 10.3390/biomedicines12122781, PMID: 39767686 PMC11673227

[ref27] Grommes J. Soehnlein O. (2011). Contribution of neutrophils to acute lung injury. Mol. Med. 17, 293–307. doi: 10.2119/molmed.2010.00138, PMID: 21046059 PMC3060975

[ref28] Haak B. W. Littmann E. R. Chaubard J. L. Pickard A. J. Fontana E. Adhi F. . (2018). Impact of gut colonization with butyrate-producing microbiota on respiratory viral infection following Allo-HCT. Blood 131, 2978–2986. doi: 10.1182/blood-2018-01-828996, PMID: 29674425 PMC6024637

[ref29] Haak B. W. Westendorp W. F. van Engelen T. S. R. Brands X. Brouwer M. C. Vermeij J.-D. . (2021). Disruptions of anaerobic gut bacteria are associated with stroke and post-stroke infection: a prospective case–control study. Transl. Stroke Res. 12, 581–592. doi: 10.1007/s12975-020-00863-4, PMID: 33052545 PMC8213601

[ref30] Hakoupian M. Ferino E. Jickling G. C. Amini H. Stamova B. Ander B. P. . (2021). Bacterial lipopolysaccharide is associated with stroke. Sci. Rep. 11:6570. doi: 10.1038/s41598-021-86083-8, PMID: 33753837 PMC7985504

[ref31] Hammond N. E. Myburgh J. Seppelt I. Garside T. Vlok R. Mahendran S. . (2022). Association between selective decontamination of the digestive tract and in-hospital mortality in intensive care unit patients receiving mechanical ventilation: a systematic review and meta-analysis. JAMA 328, 1922–1934. doi: 10.1001/jama.2022.19709, PMID: 36286098 PMC9607997

[ref32] Hediyal T. A. Vichitra C. Anand N. Bhaskaran M. Essa S. M. Kumar P. . (2024). Protective effects of fecal microbiota transplantation against ischemic stroke and other neurological disorders: an update. Front. Immunol. 15:1324018. doi: 10.3389/fimmu.2024.1324018, PMID: 38449863 PMC10915229

[ref33] Hou S. Yu J. Li Y. Zhao D. Zhang Z. (2025). Advances in fecal microbiota transplantation for gut dysbiosis-related diseases. Adv. Sci. 12:197. doi: 10.1002/advs.202413197, PMID: 40013938 PMC11967859

[ref34] Huang Y. Mao K. Chen X. Sun M. Kawabe T. Li W. . (2018). S1P-dependent interorgan trafficking of group 2 innate lymphoid cells supports host defense. Science 359, 114–119. doi: 10.1126/science.aam5809, PMID: 29302015 PMC6956613

[ref35] Human Microbiome Project Consortium (2012a). Structure, function and diversity of the healthy human microbiome. Nature 486, 207–214. doi: 10.1038/nature11234, PMID: 22699609 PMC3564958

[ref36] Human Microbiome Project Consortium (2012b). A framework for human microbiome research. Nature 486, 215–221. doi: 10.1038/nature11209 22699610 PMC3377744

[ref37] Janssen R. Van Workum F. Baranov N. Blok H. Ten Oever J. Kolwijck E. . (2021). Selective decontamination of the digestive tract to prevent postoperative pneumonia and anastomotic leakage after esophagectomy: a retrospective cohort study. Antibiotics 10:43. doi: 10.3390/antibiotics10010043, PMID: 33466226 PMC7824731

[ref38] Jo J. Hu C. Begum K. Wang W. Le T. M. Agyapong S. . (2023). Fecal pharmacokinetics and gut microbiome effects of oral omadacycline versus vancomycin in healthy volunteers. J. Infect. Dis. 229, 273–281. doi: 10.1093/infdis/jiad537, PMID: 38051631 PMC10786255

[ref39] Kalra L. Hodsoll J. Irshad S. Smithard D. Manawadu D. (2016). Association between nasogastric tubes, pneumonia, and clinical outcomes in acute stroke patients. Neurology 87, 1352–1359. doi: 10.1212/WNL.0000000000003151, PMID: 27566745 PMC5047037

[ref40] Kalra L. Irshad S. Hodsoll J. Simpson M. Gulliford M. Smithard D. . (2015). Prophylactic antibiotics after acute stroke for reducing pneumonia in patients with dysphagia (STROKE-INF): a prospective, cluster-randomised, open-label, masked endpoint, controlled clinical trial. Lancet 386, 1835–1844. doi: 10.1016/S0140-6736(15)00126-9, PMID: 26343840

[ref41] Katagiri S. Shiba T. Tohara H. Yamaguchi K. Hara K. Nakagawa K. . (2019). Re-initiation of Oral food intake following enteral nutrition alters Oral and gut microbiota communities. Front. Cell. Infect. Microbiol. 9:434. doi: 10.3389/fcimb.2019.00434, PMID: 31956606 PMC6951430

[ref42] Kaur G. Stein L. K. Boehme A. Liang J. W. Tuhrim S. Mocco J. . (2019). Risk of readmission for infection after surgical intervention for intracerebral hemorrhage. J. Neurol. Sci. 399, 161–166. doi: 10.1016/j.jns.2019.02.016, PMID: 30818077

[ref43] Kawai T. Ikegawa M. Ori D. Akira S. (2024). Decoding toll-like receptors: recent insights and perspectives in innate immunity. Immunity 57, 649–673. doi: 10.1016/j.immuni.2024.03.004, PMID: 38599164

[ref44] Kishore A. K. Vail A. Jeans A. R. Chamorro A. Di Napoli M. Kalra L. . (2018). Microbiological etiologies of pneumonia complicating stroke: A systematic review. Stroke 49, 1602–1609. doi: 10.1161/STROKEAHA.117.020250 29915122

[ref45] Kotlega D. Drozd A. Zembron-Lacny A. Morawin B. Ryterska K. Szczuko M. (2025). The activity of protectin DX, 17 HDHA and leukotriene B4 is correlated with interleukin-1β (IL-1β) and interleukin-1 receptor antagonist (IL-1Ra) in the early subacute phase of stroke. Int. J. Mol. Sci. 26:9088. doi: 10.3390/ijms26189088, PMID: 41009650 PMC12471189

[ref46] Kotlyarov S. (2022). Role of short-chain fatty acids produced by gut microbiota in innate lung immunity and pathogenesis of the heterogeneous course of chronic obstructive pulmonary disease. Int. J. Mol. Sci. 23:4768. doi: 10.3390/ijms23094768, PMID: 35563159 PMC9099629

[ref47] Labeit B. Michou E. Hamdy S. Trapl-Grundschober M. Suntrup-Krueger S. Muhle P. . (2023). The assessment of dysphagia after stroke: state of the art and future directions. Lancet Neurol. 22, 858–870. doi: 10.1016/S1474-4422(23)00153-9, PMID: 37596008

[ref48] Le J. Kulatheepan Y. Jeyaseelan S. (2023). Role of toll-like receptors and nod-like receptors in acute lung infection. Front. Immunol. 14:1249098. doi: 10.3389/fimmu.2023.1249098, PMID: 37662905 PMC10469605

[ref49] Leahy R. G. Serio A. W. Wright K. Traczewski M. M. Tanaka S. K. (2022). Activity of omadacycline in vitro against clostridioides difficile and preliminary efficacy assessment in a hamster model of *C. difficile*-associated diarrhoea. J. Glob. Antimicrob. Resist. 30, 96–99. doi: 10.1016/j.jgar.2022.04.019, PMID: 35500838

[ref50] Li Z. Gu M. Sun H. Chen X. Zhou J. Zhang Y. (2023). The potential of gut microbiota in prediction of stroke-associated pneumonia. Brain Sci. 13:1217. doi: 10.3390/brainsci13081217, PMID: 37626573 PMC10452830

[ref51] Li Z. Li Y. Sun Q. Wei J. Li B. Qiu Y. . (2022). Targeting the pulmonary microbiota to fight against respiratory diseases. Cells 11:916. doi: 10.3390/cells11050916, PMID: 35269538 PMC8909000

[ref52] Li C. Lu F. Chen J. Ma J. Xu N. (2022). Probiotic supplementation prevents the development of ventilator-associated pneumonia for mechanically ventilated ICU patients: a systematic review and network meta-analysis of randomized controlled trials. Front. Nutr. 9:156. doi: 10.3389/fnut.2022.919156, PMID: 35879981 PMC9307490

[ref53] Li T. Su X. Lu P. Kang X. Hu M. Li C. . (2023). Bone marrow mesenchymal stem cell-derived Dermcidin-containing Migrasomes enhance LC3-associated phagocytosis of pulmonary macrophages and protect against post-stroke pneumonia. Adv Sci (Weinh) 10:e2206432. doi: 10.1002/advs.202206432, PMID: 37246283 PMC10401184

[ref54] Lidetu T. Muluneh E. K. Wassie G. T. (2023). Incidence and predictors of aspiration pneumonia among stroke patients in western Amhara region, north-West Ethiopia: a retrospective follow up study. Int J Gen Med 16, 1303–1315. doi: 10.2147/IJGM.S400420, PMID: 37089139 PMC10115200

[ref55] Lin T. Jiang D. Chen W. Lin J. S. Zhang X. Chen C. . (2023). Trained immunity induced by high-salt diet impedes stroke recovery. EMBO Rep. 24:e57164. doi: 10.15252/embr.202357164, PMID: 37965920 PMC10702837

[ref56] Liu H. Bai C. Xian F. Liu S. Long C. Hu L. . (2022). A high-calorie diet aggravates LPS-induced pneumonia by disturbing the gut microbiota and Th17/treg balance. J. Leukoc. Biol. 112, 127–141. doi: 10.1002/JLB.3MA0322-458RR, PMID: 35638590

[ref57] Liu Z. Y. Wei L. Ye R.-C. Chen J. Nie D. Zhang G. . (2022). Reducing the incidence of stroke-associated pneumonia: an evidence-based practice. BMC Neurol. 22:297. doi: 10.1186/s12883-022-02826-8, PMID: 35953801 PMC9367053

[ref58] Lloyd-Price J. Abu-Ali G. Huttenhower C. (2016). The healthy human microbiome. Genome Med. 8:51. doi: 10.1186/s13073-016-0307-y, PMID: 27122046 PMC4848870

[ref59] Luo J. Chen Y. Tang G. Li Z. Yang X. Shang X. . (2022). Gut microbiota composition reflects disease progression, severity and outcome, and dysfunctional immune responses in patients with hypertensive intracerebral hemorrhage. Front. Immunol. 13:869846. doi: 10.3389/fimmu.2022.869846, PMID: 36439158 PMC9699794

[ref60] Maeda K. Koga T. Akagi J. (2016). Tentative nil per os leads to poor outcomes in older adults with aspiration pneumonia. Clin. Nutr. 35, 1147–1152. doi: 10.1016/j.clnu.2015.09.011, PMID: 26481947

[ref61] Mao J. Li Y. Feng S. Liu X. Tian Y. Bian Q. . (2020). Bufei jianpi formula improves mitochondrial function and suppresses mitophagy in skeletal muscle via the adenosine monophosphate-activated protein kinase pathway in chronic obstructive pulmonary disease. Front. Pharmacol. 11:587176. doi: 10.3389/fphar.2020.587176, PMID: 33390958 PMC7773703

[ref62] Merenstein D. J. Tancredi D. J. Karl J. P. Krist A. H. Lenoir-Wijnkoop I. Reid G. . (2024). Is there evidence to support probiotic use for healthy people? Adv. Nutr. 15:100265. doi: 10.1016/j.advnut.2024.100265, PMID: 38977065 PMC11342770

[ref63] Mohapatra S. Pathi B. K. Mohapatra I. Singh N. Sahoo J. P. Das N. K. . (2024). Bacteriological profile of patients with stroke-associated pneumonia and antimicrobial susceptibility of pathogens: a cross-sectional study. Cureus 16:e74150. doi: 10.7759/cureus.74150, PMID: 39712707 PMC11663042

[ref64] Moura I. B. Buckley A. M. Ewin D. Shearman S. Clark E. Wilcox M. H. . (2019). Omadacycline gut microbiome exposure does not induce *Clostridium difficile* proliferation or toxin production in a model that simulates the proximal, medial, and distal human colon. Antimicrob. Agents Chemother. 63:e01581-18. doi: 10.1128/AAC.01581-18, PMID: 30455242 PMC6355569

[ref65] Muñoz K. A. Ulrich R. J. Vasan A. K. Sinclair M. Wen P.-C. Holmes J. R. . (2024). A gram-negative-selective antibiotic that spares the gut microbiome. Nature 630, 429–436. doi: 10.1038/s41586-024-07502-0, PMID: 38811738 PMC12135874

[ref66] Narayana J. K. Aliberti S. Mac Aogáin M. Jaggi T. K. Ali N. A. B. M. Ivan F. X. . (2023). Microbial dysregulation of the gut-lung axis in bronchiectasis. Am. J. Respir. Crit. Care Med. 207, 908–920. doi: 10.1164/rccm.202205-0893OC, PMID: 36288294 PMC10111978

[ref67] Özçam M. Lynch S. V. (2024). The gut-airway microbiome axis in health and respiratory diseases. Nat. Rev. Microbiol. 22, 492–506. doi: 10.1038/s41579-024-01048-8, PMID: 38778224 PMC12051635

[ref68] Palleja A. Mikkelsen K. H. Forslund S. K. Kashani A. Allin K. H. Nielsen T. . (2018). Recovery of gut microbiota of healthy adults following antibiotic exposure. Nat. Microbiol. 3, 1255–1265. doi: 10.1038/s41564-018-0257-9, PMID: 30349083

[ref69] Park J. Kim M. Kang S. G. Jannasch A. H. Cooper B. Patterson J. . (2015). Short-chain fatty acids induce both effector and regulatory T cells by suppression of histone deacetylases and regulation of the mTOR–S6K pathway. Mucosal Immunol. 8, 80–93. doi: 10.1038/mi.2014.44, PMID: 24917457 PMC4263689

[ref70] Perdijk O. Azzoni R. Marsland B. J. (2024). The microbiome: an integral player in immune homeostasis and inflammation in the respiratory tract. Physiol. Rev. 104, 835–879. doi: 10.1152/physrev.00020.2023, PMID: 38059886

[ref8] Porcari S. Benech N. Valles-Colomer M. Segata N. Gasbarrini A. Cammarota G. . (2023). Key determinants of success in fecal microbiota transplantation: from microbiome to clinic. Cell Host Microbe 31, 712–733. doi: 10.1016/j.chom.2023.03.020 37167953

[ref71] Prame Kumar K. McKay L. D. Nguyen H. Kaur J. Wilson J. L. Suthya A. R. . (2025). Sympathetic-mediated intestinal cell death contributes to gut barrier impairment after stroke. Transl. Stroke Res. 16, 280–298. doi: 10.1007/s12975-023-01211-y, PMID: 38030854 PMC11976816

[ref72] Rahimi A. Qaisar S. A. Janeh T. Karimpour H. Darbandi M. Moludi J. (2024). Clinical trial of the effects of postbiotic supplementation on inflammation, oxidative stress, and clinical outcomes in patients with CVA. Sci. Rep. 14:24021. doi: 10.1038/s41598-024-76153-y, PMID: 39402150 PMC11473548

[ref73] Samary C. S. Ramos A. B. Maia L. A. Rocha N. N. Santos C. L. Magalhães R. F. . (2018). Focal ischemic stroke leads to lung injury and reduces alveolar macrophage phagocytic capability in rats. Crit. Care 22:249. doi: 10.1186/s13054-018-2164-0, PMID: 30290827 PMC6173845

[ref74] Sanders M. Beringer P. Immunomodulatory activity of omadacycline in vitro and in a murine model of acute lung injury. mSphere 2024, 9:e00671. doi: 10.1128/msphere.00671-24, PMID: 39475317 PMC11580420

[ref75] Scott N. A. Andrusaite A. Andersen P. Lawson M. Alcon-Giner C. Leclaire C. . (2018). Antibiotics induce sustained dysregulation of intestinal T cell immunity by perturbing macrophage homeostasis. Sci. Transl. Med. 10:eaao4755. doi: 10.1126/scitranslmed.aao4755, PMID: 30355800 PMC6548564

[ref76] Sheerah H. A. Eshak E. S. Cui R. Imano H. Iso H. Tamakoshi A. . (2018). Relationship between dietary vitamin D and deaths from stroke and coronary heart disease: the Japan collaborative cohort study. Stroke 49, 454–457. doi: 10.1161/STROKEAHA.117.019417, PMID: 29311267

[ref77] Shen Z. Luo W. Tan B. Nie K. Deng M. Wu S. . (2022). *Roseburia intestinalis* stimulates TLR5-dependent intestinal immunity against crohn’s disease. EBioMedicine 85:104285. doi: 10.1016/j.ebiom.2022.104285, PMID: 36182776 PMC9526137

[ref78] Shen H. Zhan B. (2020). Effect of vitamin E on stroke-associated pneumonia. J. Int. Med. Res. 48:300060520949657. doi: 10.1177/0300060520949657, PMID: 32910689 PMC7488915

[ref79] Shichita T. Sugiyama Y. Ooboshi H. Sugimori H. Nakagawa R. Takada I. . (2009). Pivotal role of cerebral interleukin-17-producing gammadeltaT cells in the delayed phase of ischemic brain injury. Nat. Med. 15, 946–950. doi: 10.1038/nm.1999, PMID: 19648929

[ref80] Shim R. Wong C. H. Y. (2018). Complex interplay of multiple biological systems that contribute to post-stroke infections. Brain Behav. Immun. 70, 10–20. doi: 10.1016/j.bbi.2018.03.019, PMID: 29571897

[ref81] Siracusa F. Schaltenberg N. Kumar Y. Lesker T. R. Steglich B. Liwinski T. . (2023). Short-term dietary changes can result in mucosal and systemic immune depression. Nat. Immunol. 24, 1473–1486. doi: 10.1038/s41590-023-01587-x, PMID: 37580603 PMC10457203

[ref82] Smith C. J. Emsley H. C. Udeh C. T. Vail A. Hoadley M. E. Rothwell N. J. . (2012). Interleukin-1 receptor antagonist reverses stroke-associated peripheral immune suppression. Cytokine 58, 384–389. doi: 10.1016/j.cyto.2012.02.016, PMID: 22445501

[ref83] Smith C. J. Hulme S. Vail A. Heal C. Parry-Jones A. R. Scarth S. . (2018). SCIL-STROKE (subcutaneous interleukin-1 receptor antagonist in ischemic stroke). Stroke 49, 1210–1216. doi: 10.1161/STROKEAHA.118.020750, PMID: 29567761

[ref84] Smith C. J. Kishore A. K. Vail A. Chamorro A. Garau J. Hopkins S. J. . (2015). Diagnosis of stroke-associated pneumonia: recommendations from the pneumonia in stroke consensus group. Stroke 46, 2335–2340. doi: 10.1161/STROKEAHA.115.009617 26111886

[ref85] Sonomoto K. Song R. Eriksson D. Hahn A. M. Meng X. Lyu P. . (2023). High-fat-diet-associated intestinal microbiota exacerbates psoriasis-like inflammation by enhancing systemic γδ T cell IL-17 production. Cell Rep. 42:112713. doi: 10.1016/j.celrep.2023.112713, PMID: 37421628 PMC10391630

[ref86] Sørensen R. T. Rasmussen R. S. Overgaard K. Lerche A. Johansen A. M. Lindhardt T. (2013). Dysphagia screening and intensified oral hygiene reduce pneumonia after stroke. J. Neurosci. Nurs. 45, 139–146. doi: 10.1097/JNN.0b013e31828a412c, PMID: 23636069

[ref87] Stanley D. Mason L. J. Mackin K. E. Srikhanta Y. N. Lyras D. Prakash M. D. . (2016). Translocation and dissemination of commensal bacteria in post-stroke infection. Nat. Med. 22, 1277–1284. doi: 10.1038/nm.4194, PMID: 27694934

[ref88] Stanley D. Moore R. J. Wong C. H. Y. (2018). An insight into intestinal mucosal microbiota disruption after stroke. Sci. Rep. 8:568. doi: 10.1038/s41598-017-18904-8, PMID: 29330443 PMC5766598

[ref89] Swidsinski A. Loening-Baucke V. Krüger M. Kirsch S. (2012). Central nervous system and the colonic bioreactor: analysis of colonic microbiota in patients with stroke unravels unknown mechanisms of the host defense after brain injury. Intest. Res. 10:332. doi: 10.5217/ir.2012.10.4.332

[ref90] Tan C. Wu Q. Wang H. Gao X. Xu R. Cui Z. . (2021). Dysbiosis of gut microbiota and short-chain fatty acids in acute ischemic stroke and the subsequent risk for poor functional outcomes. J. Parenter. Enter. Nutr. 45, 518–529. doi: 10.1002/jpen.1861, PMID: 32473086 PMC8048557

[ref91] Tedelind S. Westberg F. Kjerrulf M. Vidal A. (2007). Anti-inflammatory properties of the short-chain fatty acids acetate and propionate: a study with relevance to inflammatory bowel disease. World J. Gastroenterol. 13:2826. doi: 10.3748/wjg.v13.i20.2826 17569118 PMC4395634

[ref92] Teuschl Y. Trapl M. Ratajczak P. Matz K. Dachenhausen A. Brainin M. (2018). Systematic dysphagia screening and dietary modifications to reduce stroke-associated pneumonia rates in a stroke-unit. PLoS One 13:e0192142. doi: 10.1371/journal.pone.0192142, PMID: 29389984 PMC5794132

[ref93] Thibeault C. Suttorp N. Opitz B. (2021). The microbiota in pneumonia: from protection to predisposition. Sci. Transl. Med. 13:501. doi: 10.1126/scitranslmed.aba0501, PMID: 33441423

[ref94] Touch S. Godefroy E. Rolhion N. Danne C. Oeuvray C. Straube M. . (2022). Human CD4+CD8α+ tregs induced by *Faecalibacterium prausnitzii* protect against intestinal inflammation. JCI Insight 7:e154722. doi: 10.1172/jci.insight.154722, PMID: 35536673 PMC9309064

[ref95] Vinolo M. A. R. Rodrigues H. G. Hatanaka E. Sato F. T. Sampaio S. C. Curi R. (2011). Suppressive effect of short-chain fatty acids on production of proinflammatory mediators by neutrophils. J. Nutr. Biochem. 22, 849–855. doi: 10.1016/j.jnutbio.2010.07.009, PMID: 21167700

[ref96] Wang L. Cai Y. Garssen J. Henricks P. A. J. Folkerts G. Braber S. (2023). The bidirectional gut–lung axis in chronic obstructive pulmonary disease. Am. J. Respir. Crit. Care Med. 207, 1145–1160. doi: 10.1164/rccm.202206-1066TR, PMID: 36883945 PMC10161745

[ref97] Wang R. Gan C. Mao R. Chen Y. Yan R. Li G. . (2024). Rat models of postintracerebral hemorrhage pneumonia induced by nasal inoculation with *Klebsiella pneumoniae* or intratracheal inoculation with LPS. Front. Immunol. 15:1477902. doi: 10.3389/fimmu.2024.1477902, PMID: 39845950 PMC11750689

[ref98] Wang H. Song W. Wu Q. Gao X. Li J. Tan C. . (2021). Fecal transplantation from db/db mice treated with sodium butyrate attenuates ischemic stroke injury. Microbiol. Spectr. 9:e0004221. doi: 10.1128/Spectrum.00042-21, PMID: 34612696 PMC8510264

[ref99] Wang J. Zhang H. He J. Xiong X. (2022). The role of the gut microbiota in the development of ischemic stroke. Front. Immunol. 13:845243. doi: 10.3389/fimmu.2022.845243, PMID: 35418976 PMC8995494

[ref100] Wen S. W. Wong C. H. Y. (2017). An unexplored brain-gut microbiota axis in stroke. Gut Microbes 8, 601–606. doi: 10.1080/19490976.2017.1344809, PMID: 28640714 PMC5730388

[ref101] Westendorp W. F. Vermeij J.-D. Zock E. Hooijenga I. J. Kruyt N. D. Bosboom H. J. L. W. . (2015). The preventive antibiotics in stroke study (PASS): a pragmatic randomised open-label masked endpoint clinical trial. Lancet (Lond Engl) 385, 1519–1526. doi: 10.1016/S0140-6736(14)62456-9, PMID: 25612858

[ref102] Wilson A. S. Koller K. R. Ramaboli M. C. Nesengani L. T. Ocvirk S. Chen C. . (2020). Diet and the human gut microbiome: an international review. Dig. Dis. Sci. 65, 723–740. doi: 10.1007/s10620-020-06112-w, PMID: 32060812 PMC7117800

[ref103] Wu T. Li H. Su C. Xu F. Yang G. Sun K. . (2020). Microbiota-derived short-chain fatty acids promote LAMTOR2-mediated immune responses in macrophages. Msystems 5, e00587–e00520. doi: 10.1128/mSystems.00587-20, PMID: 33144310 PMC7646525

[ref104] Wypych T. Wickramasinghe L. Marsland B. (2019). The influence of the microbiome on respiratory health. Nat. Immunol. 20, 1279–1290. doi: 10.1038/s41590-019-0451-9, PMID: 31501577

[ref105] Xia G.-H. Zhang M.-S. Wu Q.-H. Wang H.-D. Zhou H.-W. He Y. . (2021). Dysbiosis of gut microbiota is an independent risk factor of stroke-associated pneumonia: a chinese pilot study. Front. Cell. Infect. Microbiol. 11:715475. doi: 10.3389/fcimb.2021.715475, PMID: 34414134 PMC8369370

[ref106] Xie X. Wang L. Dong S. Ge S. Zhu T. (2023). Immune regulation of the gut-brain axis and lung-brain axis involved in ischemic stroke. Neural Regen. Res. 19, 519–528. doi: 10.4103/1673-5374.380869, PMID: 37721279 PMC10581566

[ref107] Xie B. Zhang Y. Han M. Wang M. Yu Y. Chen X. . (2023). Reversal of the detrimental effects of social isolation on ischemic cerebral injury and stroke-associated pneumonia by inhibiting small intestinal γδ T-cell migration into the brain and lung. J. Cereb. Blood Flow Metab. 43, 1267–1284. doi: 10.1177/0271678X231167946, PMID: 37017434 PMC10369145

[ref108] Yamashiro K. Tanaka R. Urabe T. Ueno Y. Yamashiro Y. Nomoto K. . (2017). Gut dysbiosis is associated with metabolism and systemic inflammation in patients with ischemic stroke. PLoS One 12:e0171521. doi: 10.1371/journal.pone.0171521, PMID: 28166278 PMC5293236

[ref109] Yin J. Liao S.-X. He Y. Wang S. Xia G.-H. Liu F.-T. . (2015). Dysbiosis of gut microbiota with reduced trimethylamine-N-oxide level in patients with large-artery atherosclerotic stroke or transient ischemic attack. J. Am. Heart Assoc. 4:e002699. doi: 10.1161/JAHA.115.002699, PMID: 26597155 PMC4845212

[ref110] Yuen J. K. Luk J. K. H. Chan T.-C. Shea Y.-F. Chu S. T. Bernacki R. . (2022). Reduced pneumonia risk in advanced dementia patients on careful hand feeding compared with nasogastric tube feeding. J. Am. Med. Dir. Assoc. 23, 1541–1547.e2. doi: 10.1016/j.jamda.2022.03.011, PMID: 35489380

[ref111] Zhang H. Huang Y. Li X. Han X. Hu J. Wang B. . (2021). Dynamic process of secondary pulmonary infection in mice with intracerebral hemorrhage. Front. Immunol. 12:767155. doi: 10.3389/fimmu.2021.767155, PMID: 34868020 PMC8639885

[ref112] Zhang J. Ling L. Xiang L. Li W. Bao P. Yue W. (2024). Role of the gut microbiota in complications after ischemic stroke. Front. Cell. Infect. Microbiol. 14:581. doi: 10.3389/fcimb.2024.1334581, PMID: 38644963 PMC11026644

[ref113] Zhang H. Sheng S. Li C. Bao X. Zhao L. Chen J. . (2024). Mucosal immunization with the lung lactobacillus-derived amphiphilic exopolysaccharide adjuvanted recombinant vaccine improved protection against *P. aeruginosa* infection. PLoS Pathog. 20:e1012696. doi: 10.1371/journal.ppat.1012696, PMID: 39556597 PMC11611261

[ref114] Zharikova A. A. Andrianova N. V. Silachev D. N. Nebogatikov V. O. Pevzner I. B. Makievskaya C. I. . (2025). Analysis of the brain transcriptome, microbiome and metabolome in ketogenic diet and experimental stroke. Brain Behav. Immun. 123, 571–585. doi: 10.1016/j.bbi.2024.10.004, PMID: 39378970

